# TICU-Feedback-Tool: development and pilot application of a questionnaire to assess performance in tele-intensive care collaborations

**DOI:** 10.1186/s12913-025-12565-4

**Published:** 2025-03-20

**Authors:** Franziska Lezius, Karin Steinecke, Anne Herholz, Stephen Schüürhuis, Andreas Edel, Michaela Niebank, Nicolai Andrees, Claudia D. Spies, Björn Weiss, U START TICU consensus group, Mohammed Al-Ashwal, Alexandra Becker, Friedrich Borchers, Martina Gaßner, Michele Ocken, Clemens Hoffmann, Fathima Paruk, Kay Rumschüßel, Doreen Fuhl, Stefan Heidemann, Hans-Joachim Janssen, Frank Trebus, Tobias Klöpper, Christoph Büttner, Christoph Bauhuis

**Affiliations:** 1 Department of Anesthesiology and Intensive Care Medicine, Charité – Universitätsmedizin Berlin, Corporate Member of Freie Universität Berlin and Humboldt Universität zu Berlin, Augustenburger Platz 1, Berlin, 13353 Germany; 2 Department of Infectious Diseases, Klinik Bavaria, Kreischa, Germany; 3 Institute of Biometry and Clinical Epidemiology, Charité – Universitätsmedizin Berlin, Corporate Member of Freie Universität Berlin and Humboldt-Universität zu Berlin, Berlin, Germany; 4 Department of Anesthesiology and Intensive Care Medicine, Universitätsklinikum Ulm, Ulm, Germany; 5 Centre for Biological Threats and Special Pathogens (ZBS), Robert Koch Institute, Berlin, Germany

**Keywords:** Telemedicine, Intensive care unit, Feedback, Quality

## Abstract

**Background:**

Telemedicine is a suitable vehicle to facilitate collaboration among hospitals across borders, with the COVID-19 pandemic paving the way for rapidly growing tele-intensive care (TICU) networks, aiming to improve quality of care. Hitherto there are no validated instruments to assess and evaluate performance in international TICU collaboration.

**Methods:**

We conducted a prospective, structured survey development study with a single-step online expert consensus approach and a pilot application.

**Results:**

We propose a 26-indicator TICU-Feedback-Tool assessing user-friendliness, subjective benefit and usability, acceptance and potential for improvement in TICU networks. The instrument is suitable for self-reporting by online questionnaire.

**Conclusion:**

We suggest a pilot version of a feedback questionnaire for quality management in (inter-)national TICU networks that will be subject to revisions in the future.

**Supplementary Information:**

The online version contains supplementary material available at 10.1186/s12913-025-12565-4.

## Introduction

Telemedicine has evolved quickly over the past decade. It has been established in various areas ranging from clinical applications to grant access to specialty care in remote areas [1, 2] to tele-educational training programs to share medical expertise with colleagues [3, 4]. The Covid-19 pandemic tremendously accelerated the needs in combined effort in the management of critically ill patients in intensive care units (ICU) worldwide [5, 6]. Tele-intensive care units (TICU) have demonstrated to be a suitable vehicle to facilitate collaboration regionally [7] as well as across borders [8, 9] aiming to improve the quality in critical care.

To ensure satisfying standards in telemedicine (e.g. technically and clinically), recurrent assessments of the tele-medical services are essential to enforce progress. A variety of feedback instruments proved useful in different tele-medical contexts [10, 11]. The instruments evaluate different areas of interest in tele-medical collaboration i.e. usability, satisfaction, acceptance and process. The design of these questionnaires is diverse including Yes/No-questions, scales, multiple choice and open-ended questions. A wide variety of specialties including surgical, medical and emergency services is covered in in- and outpatient settings. The questionnaires address different participants predominantly patients and caregivers or medical personnel and are developed for use in various countries internationally. Yip et al. [12], Bakken et al. [13] and Buysse et al. [14] for example suggested questionnaires addressing patients with diabetes in outpatient care to rate the satisfaction with telemedicine, its usefulness, the communication with peers and professionals via telemedical devices as well as telemonitoring health effects in their treatment. Morgan et al. [15] developed a survey to evaluate the satisfaction with telemedicine of patients and their caregivers in a rural memory clinic population whereas Parmanto et al. [16] proposed a questionnaire to assess usability of telehealth implementation and services addressing both clinicians and their clients.

Tele- intensive care networks, however, may differ from other tele-medical settings in multiple aspects. Technical performance in intensive care units can be challenging due to unstable access to the internet or interference with a multitude of technical (medical) applications. Benefit and acceptance are typically rated by medical staff as patients are often unable to self-report. Collaboration may be time critical due to rapid changes in medical condition. Shortage of staff and heavy workload demand a comprehensive but concise questionnaire, aiming to cover the most crucial areas of tele-intensive care collaboration.

Therefore, we suggest a TICU-Feedback-Tool as an instrument to assess (a) interprofessional user-friendliness, (b) subjective benefit and usability, (c) acceptance and potential areas of improvements in TICU networks.

## Methods

We carried out a prospective, structured survey development study with a single-step online consensus approach and a pilot application within our international TICU network from August 16, 2023 to November 2, 2023. The study was approved by the local ethics committee of Charité – Universitätsmedizin Berlin (registered under No. EA2/163/23).

The proposed tool roots in an extended version of a feedback instrument (unpublished) used in a national TICU network study (clinical trials’ identifier: NCT03671447). The initial extended feedback instrument was developed by a team of specialist intensivists experienced in TICU collaboration and specialists for public health with expertise in survey development aiming to assess the technical and medical performance in TICU networks in general as well as its use and significance in crisis management during the COVID-19 pandemic.

**Fig. 1:**
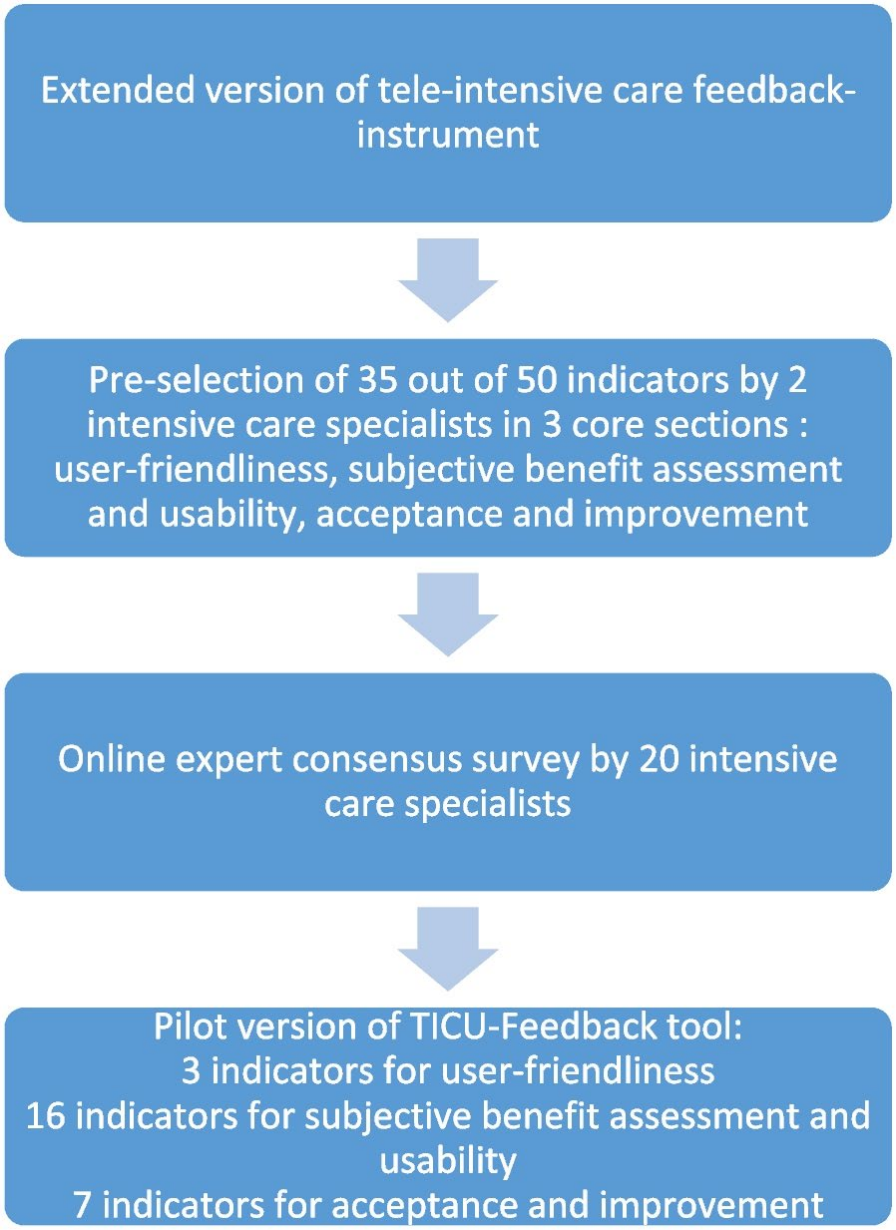
Development of TICU-Feedback-Tool

The following steps were conducted to develop the TICU-Feedback-Tool (Fig. 1): pre-selection of indicators by two intensive care specialists (FL, KS) from extended 50-item feedback instrument, online expert consensus survey by 20 intensive care specialists to rate the core questions regarding “importance” and “reliability for self-reporting” (Table S1, supplement 1) and subsequently pilot application of the tool completed by 20 participants from our international TICU network.

In the pre-selection of indicators, we excluded (i) all indicators that were specific to the setting of the initial regional study carried out during the COVID-19 pandemic namely indicators referring to crisis management or the use of telemedicine over the course of the study period and (ii) a few indicators that were of explicit interest in regional rather than international networks e.g. transfers of patients between hospitals. All indicators i.e. 35 that were considered to be of general interest for TICU collaboration were included in the consensus survey. Categorizations of the indicators was preserved therefore identical to the extended questionnaire except for one additional category referring to crisis management during the COVID-19 pandemic which we excluded entirely.

In the consensus survey the participating intensive care specialists were given two options to rate the “importance” and the “reliability for true self-reporting” of each indicator as either “high” or “low”. Before entering the survey, participants received a comprehensive written instruction on the purpose of the questionnaire and the two features for assessment (“importance” and”reliability for true self-reporting”) (supplement 2: instructions for participants). The aim was to find (i) the most important questions to evaluate satisfaction with the tele-medical consultations and the technical user friendliness and (ii) to decide whether questions will be answered truthfully. The threshold to include a question in the pilot version of the tool was defined by 75%-agreement for “importance high”. “Reliability for true self-reporting” was rated “high versus low” for all indicators (Table S1, supplement 1) but prioritizing “importance” of an indicator over “reliability for true self-reporting” in this pilot version of the assessment instrument, exclusion of indicators was solely based on importance.

Subsequently following the consensus process we carried out a pilot application of the tool within our international network addressing all medical staff i.e. physicians and nurses regularly collaborating in TICU consultations. Medical personnel from eight different hospitals in four countries were invited to participate. The principal aim of the pilot application was the psychometric evaluation of the scale.

The consensus survey as well as the pilot application were conducted as an online questionnaire in English language using the statistical survey web app LimeSurvey. As language proficiency in English was ensured all participants received the same survey. Respondents’ consent was obtained by actively agreeing on study and data protection information before entering the questionnaire.

### Statistical analysis

Data was analyzed with SPSS Statistics 27. Due to the exploratory nature of this survey, statistical analysis was conducted using methods of descriptive statistics.

In the consensus survey, we report the absolute and relative frequencies of participants who rated the pre-selected items as highly important, as well as those who rated the 'reliability for true self-reporting' as high. Additionally, for all core sections, we present the mean percentages of participants who rated the 'reliability for true self-reporting' as high across all indicators within each core section as a summary measure for each core section.

After the consensus survey, the pilot version of the feedback tool was evaluated in a pilot application. For all selected indicators, we report absolute and relative frequencies for each category of the underlying 5-point Likert scale. To get a preliminary understanding of the feedback tool’s psychometric properties, we computed (unstandardized) Cronbach’s alpha, including 95% confidence intervals, as a measure of internal consistency to evaluate the correlation between items within the same subsection. Cronbach’s alpha was interpreted as acceptable for values greater than 0.7 and good for values greater than 0.8. To assess the extent to which each item measures the underlying core section, separability (corrected item-total correlation) was calculated for each item [17–21]. The cut off for separability was set at 0.3 differentiating between high versus low correlation of each item with the respective category [20].

## Results

### Survey development

All forty-six intensivists within our network who had significant experience in telemedicine and were board certified specialists in their country, were invited to participate. 20 (43%) of them completed the consensus questionnaire. The 20 participating intensivists were from nine different hospitals; there was one specialist from South Africa and 19 from Germany, eight (40%) were female. Seventeen (85%) intensive care specialists had a professional medical background in anesthesiology and three in internal medicine.

In total 24 (71%) out of 35 indicators in all categories were selected by the consensus survey, meeting the prerequisite of a ≥ 75% threshold for “importance high” (Table S1, supplement 1). Table S2 (supplement 3) shows all indicators finally included in the pilot version of the TICU-Feedback-Tool. All but one question i.e. “What points would you like to change about the tele-medical visits for further improvement?” are rated by a 5-point Likert scale ranging from “strongly disagree” to “strongly agree”.

The first core section “user-friendliness” is entirely derived from the well-known “System usability scale (SUS)” [22] to allow comparability with other tele-medical devices and prior studies. In this first category, 7 (70%) out of 10 questions were rated with less than 75% approval for “importance high”. The mean percentage for “reliability for true self reporting high” across all indicators in the core section “user-friendliness” was 72% (SD 14.0%).

The second core category “subjective benefit assessment and usability” comprises six sub-sections namely subjective benefit assessment: benefit for patients and benefit for medical staff, trust and working relationship, interaction with patients, technical performance and overall impression. In this core section, 16 (100%) out of 16 indicators were consented with ≥ 75%. The mean percentage for “reliability for true self reporting high” for all indicators in this respective category was 76.9% (SD 15.7%).

In the third core category “acceptance and improvement”, five (56%) out of nine indicators from the following two sub-sections “attitude of the "users" towards tele-medical rounds” and “potential for improvement” were approved by the consensus survey. Despite the fact that four indicators were rejected by the consensus process, only two (22%) questions were excluded from the pilot version of the tool. The two following questions in this category were not approved by the consensus survey with the required ≥ 75% of agreement of high importance, but were nevertheless included in the pilot version of the TICU-Feedback-Tool:

• I have ethical concerns about tele-medical visits
• I have data protection concerns about tele-medical visits

This third core section “acceptance and improvement” showed a mean percentage of 67% (SD 9.1%) for “reliability for true self reporting high”.

### Pilot application

As a pilot application, the questionnaire was completed by 20 participants namely medical personnel from eight different hospitals in four countries collaborating via regular tele-medical consultations within our international TICU network. Table 1 shows the results of the pilot application displayed by single indicators. All indicators i.e. 25 that are rated on a 5-point Likert scale are displayed in Table 1, the last question referring to the potential for improvement is the single open-ended question of the survey, therefore ratings of separability are not applicable. Cronbach’s alpha was analyzed by subsections with core category one “user-friendliness” (0.75, 95% CI [0.477, 0.894]) and two “subjective benefit assessment and usability” (0.85, 95% CI [0.725, 0.928]) showing results of close to 0.8 or above and subcategory three “acceptance and improvement” showing a Cronbach’s alpha of 0.51 (95% CI [0.078, 0.779]). Separability per item was calculated, with the following two indicators in subsection two, and three indicators in category three displaying results < 0.3: “If necessary, tele-medical visits were arranged at short notice” (0.26), “I was always able to address any uncertainties or treatment errors openly” (0.18), “In principle, I have a positive attitude towards tele-medical consultations using the device” (0.07), “Overall, I am satisfied with tele-medical rounds” (0) and “All in all, I think the tele-medical visits work well” (0.18).

**Table 1:** Pilot application

Indicators	Strongly disagree	Disagree	Neutral	Agree	Strongly agree	*Separability*
I think I would like to use this system/tele-medical device frequently	2 (10%)		1 (5%)	6 (30%)	11 (55%)	*0.68*
I thought the system/tele-medical device was easy to use	1 (5%)			7 (35%)	12 (60%)	*0.80*
I felt very confident using the system/tele-medical device			2 (10%)	10 (50%)	8 (40%)	*0.39*
Patient safety was improved/increased by the tele-medical visits	1 (5%)		3 (15%)	11 (55%)	5 (25%)	*0.63*
The patients' quality of care was improved by the tele-medical visits			1 (5%)	11 (55%9	8 (40%)	*0.67*
The telemedical visits positively contributed to error avoidance/error prevention in the treatment of my patients	1 (5%)		2 (10%)	14 (70%)	3 (15%)	*0.33*
I felt well supported in taking difficult decisions by tele-medical visits				11 (55%)	9 (45%)	*0.70*
There was always enough time to discuss my concerns during tele-medical visits				9 (45%)	11 (55%9	*0.37*
If necessary, telemedical visits were arranged at short notice		1 (5%)		11 (55%)	8 (40%)	*0.26*
By taking part in tele-medical visit, I was able to refresh or acquire important medical knowledge				11 (55%)	9 (45%)	*0.40*
I was always able to address any uncertainties or treatment errors openly			1 (5%)	12 (60%)	7 (45%)	*0.18*
I implemented the treatment plans as discussed in tele-medical visits			2 (10%)	14 (70%)	4 (20%)	*0.55*
The collaboration with the tele-medical specialist was always friendly and constructive				3 (15%)	17 (85%)	*0.38*
During the rounds, the tele-medical specialist treated my patients respectfully		1 (5%)	1 (5%)	2 (10%)	16 (80%)	*0.30*
My patients accepted the tele-medical specialist very well			3 (15%)	8 (40%)	9 (45%)	*0.73*
The picture quality was good and error free			1 (5%)	14 (70%)	5 (25%)	*0.68*
The sound quality was good and error-free			2 (10%)	13 (65%)	5 (25%)	*0.60*
From the technical side communication with the tele-medical physician functioned well			1 (5%)	10 (50%)	9 (45%)	*0.36*
The tele-medical rounds helped me in treating my patients			1 (5%)	9 (45%)	10 (50%)	*0.54*
In principle, I have a positive attitude towards tele-medical consultations using the device				9 (45%)	11 (55%)	*0.07*
I have ethical concerns about tele-medical visits	5 (25%)	8 (40%)	4 (20%)	2 (10%)	1 (5%)	*0.54*
I have data protection concerns about tele-medical visits	4 (20%)	7 (35%)	4 (20%)	4 (20%)	1 (5%)	*0.33*
Overall, I am satisfied with tele-medical rounds				10 (50%)	10 (50%)	*0*
All in all, I think the tele-medical visits work well				8 (40%)	12 (60%)	*0.18*
All in all, I think there is still potential for quality improvement of the tele-medical visits		2 (10%)	5 (25%)	8 (40%)	5 (25%)	*0.40*

## Discussion

Tele-intensive care networks are prospering (inter-)nationally aiming to facilitate collaboration between hospitals and to improve quality of care [4, 7, 9]. To ensure high quality tele-intensive care services and to promote continuous needs-adapted progress we propose a pilot version of a TICU-Feedback-Tool to monitor user-friendliness, subjective benefit and usability, acceptance and potential for improvement.

The tool is based on an extended feedback instrument used in a previous study of a national TICU network. Despite not being formally validated, this initial feedback instrument has proved its value specific to telemedicine in critical care settings. Standardization for international use and practicability in busy work environments, however, was needed. Pre-selection of indicators was conducted by two intensive care specialists followed by one round of an expert consensus to identify the indicators for the pilot version of the tool. In line with standard recommendations in consensus decisions we opted for a panel of experts rather than a representative sample [23]. Therefore, board certification in intensive care medicine and extensive experience in TICU collaboration was an obligation to participate in the consensus and set a sound standard to grant appropriate qualification. Contrarily, it also limited the number of experts and resulted in a rather homogenous group of participants regarding nationality and professional medical background. Whereas we chose the threshold of 75% to define consensus congruent with the majority of consensus surveys [24], the single round consensus process may have influenced the selection of indicators and subsequently the applicability for international use additionally. The response rate of 43% is in line with prior research. Response rates < 50% are often reported in consensus decisions [25] and are commonly seen in online surveys, including surveys addressing health professionals [26, 27]. Moreover, previous research suggests an adequate response rate for surveys of > 40% [28] and indicates that an increase in survey’ recipients does not generate a higher response rate [27]. Hence our response rate of 43% is acceptable.

As self-report may bias data in surveys [29] “reliability for true self-reporting” for each indicator was evaluated in the consensus survey to identify specific items potentially prone to self-report bias. We decided to base the consensus decision entirely on the importance of an indicator as the diversity of approval regarding the reliability for truthful answers would have resulted in a high reduction in indicators. Nevertheless, this expert consensus process created awareness as to which questions may be less reliable in future routine application.

The questionnaire is divided into three core categories: user-friendliness, subjective benefit assessment and usability, acceptance and improvement reflecting the most commonly used categories in telemedical questionnaires [11]. The first category i.e. “user-friendliness” is based on the well-established System Usability Scale [22]. In this category, the fewest number of indicators namely 30% was approved by the consensus survey. This section purely addresses technical aspects of the tele-medical services. Emphasizing clinical over technical aspects in TICU collaboration may be the reason for a greater reduction in indicators compared to the other two core categories. To facilitate a distinct evaluation of the user-friendliness and technical performance of our system for comparability with other tele-medical devices and prior studies a detailed analysis based on a larger group of participants is ongoing and will be reported separately in the future.

In the second core category “subjective benefit assessment and usability” all questions were confirmed by the consensus process and showed the highest agreement for truthful answers (76.9% “reliability for true self-reporting high”). Comparable indicators have been proposed in previous studies in regional TICU networks. Previous research has demonstrated positive effects of TICU-collaboration on objective outcome parameters e.g. mortality or ICU/ hospital length of stay [30–35]. As access to objective outcome parameters is very limited in international TICU networks this core category measures the clinical benefit in TICU collaboration and is in line with previous studies [36, 37].

In the last core section “acceptance and improvement” two questions addressing ethical and data protection concerns in TICU collaboration were included in the pilot version of the tool despite the fact that they were not selected by the consensus survey. The decision to grant an exception was based on the relevance of these indicators as essentials in telemedicine recommended by the World Health Organization (WHO) [38] and congruent with prior research reporting privacy concerns as a major barrier to telemedicine adoption [39]. The rather homogenous group of consensus experts may have been the reason for low rates of approval for these two indicators but their significance in an international TICU network with diverse cultural backgrounds and legal requirements is immanent.

The pilot application primarily aiming to psychometrically evaluate the scale showed rather homogenous results for all but three indicators in the third category. The two indicators exceptionally included in the tool despite not meeting the ≥ 75% consensus threshold i.e. “I have ethical concerns about tele-medical visits” and “I have data protection concerns about tele-medical visits” displayed the notably highest variance in answers, followed by “All in all, I think there is still potential for quality improvement of the tele-medical visits”. Additionally, Cronbach’s alpha of the first two core categories (0.75, 0.85) was acceptable and good and therefore comparable to Cronbach’s alpha of scales previously used in telemedicine [13–16, 40] whereas Cronbach’s alpha of the third category (0.51) is significantly lower indicating that this section may require further investigation and potential modification in future versions of the tool. For three indicators in this category separability was < 0.3 therefore showing a low correlation of each item with this specific category. Consequently, the third core section needs to be analyzed by single indicators or divided into subscales in future use of the TICU-Feedback-Tool. Summarizing the results of this core section is inadequate.

This study has several limitations specifically (i) the homogeneity of the expert consensus group, (ii) the single-round consensus approach, the mere rating of the reliability for true self-reporting, the decision to grant an exception for two indicators formally excluded by the consensus survey and the restricted number of participants of the pilot application as an obstacle to a genuine validation of the survey. While this study, with a sample of 20 participants, provided only preliminary insights into the psychometric properties of the pilot version of the feedback tool, larger future studies—following potential revisions to the feedback tool —may employ more comprehensive psychometric methods, such as factor analysis to explore the tool’s dimensionality, along with additional analyses addressing its reliability and validity.

Strengths of this study however include the structured methodological development in a prospective consensus-based approach, (i) access to several experts twinning clinical expertise with strong tele-intensive care experience (iii) an acceptable response rate and (iv) a pilot application for psychometric evaluation of the scale within our international TICU network whereas other tools are often used without any prior psychometric assessment.

In total, this pilot version of the TICU-Feedback-Tool comprises 26 out of the original 50 indicators in all categories meeting the objective to develop a comprehensive but concise instrument. The scale is convenient for easy online use. As access to objective outcome measures is very limited, technical prerequisites vary and cultural backgrounds, ethical considerations and legal requirements are manifold in diverse health care systems this tool approaches the most crucial areas to monitor quality in international TICU-collaboration. Therefore, we decided to start regular application to identify strengths and weaknesses in everyday use with the input from an expanding TICU network. The data collected from routine use may inform further refinements and psychometric analyses, aiming to improve the feedback tool’s validity over time.

## Supplementary Information

Supplementary Material 1.

Supplementary Material 2.

Supplementary Material 3.

## Data Availability

The datasets used and/or analysed during the current study are available from the corresponding author on reasonable request.

## References

[1] Sauers-Ford HS, Marcin JP, Underwood MA, Kim JH, Nicolau Y, Uy C, et al. The use of telemedicine to address disparities in access to specialist care for neonates. Telemed J E Health. 2019;25(9):775–80. 30394853 10.1089/tmj.2018.0095

[2] Boots RJ, Singh S, Terblanche M, Widdicombe N, Lipman J. Remote care by telemedicine in the ICU: many models of care can be effective. Curr Opin Crit Care. 2011;17(6):634–40. 22067879 10.1097/MCC.0b013e32834a789a

[3] Sinvani L, Delle Site C, Laumenede T, Patel V, Ardito S, Ilyas A, et al. Improving delirium detection in intensive care units: multicomponent education and training program. J Am Geriatr Soc. 2021;69(11):3249–57. 34402046 10.1111/jgs.17419

[4] Kovacevic P, Dragic S, Kovacevic T, Momcicevic D, Festic E, Kashyap R, et al. Impact of weekly case-based tele-education on quality of care in a limited resource medical intensive care unit. Crit Care. 2019;23(1):220. 31200761 10.1186/s13054-019-2494-6 PMC6567671

[5] Arabi YM, Azoulay E, Al-Dorzi HM, Phua J, Salluh J, Binnie A, et al. How the COVID-19 pandemic will change the future of critical care. Intensive Care Med. 2021;47(3):282–91. 33616696 10.1007/s00134-021-06352-y PMC7898492

[6] Van Ee SK, McKelvey H, Williams T, Shao B, Lin WT, Luu J, et al. Telemedicine Intensive Care Unit (Tele-ICU) implementation during COVID-19: a scoping review. Cureus. 2022;14(5):e25133. 35746989 10.7759/cureus.25133 PMC9206410

[7] Spies CD, Paul N, Adrion C, Berger E, Busse R, Kraufmann B, et al. Effectiveness of an intensive care telehealth programme to improve process quality (ERIC): a multicentre stepped wedge cluster randomised controlled trial. Intensive Care Med. 2023;49(2):191–204. 36645446 10.1007/s00134-022-06949-x PMC9841931

[8] Boklage E, Weiss B, Hanefeld J, Steinecke K, Jansen A, Anvarov K, et al. Telemedicine in emergency responses: reflections from a critical care telemedicine programme between Uzbekistani and German clinicians during COVID-19. BMJ Health Care Inform. 2023;30(1):e100675. 36801830 10.1136/bmjhci-2022-100675 PMC9943691

[9] Moughrabieh A, Weinert C. Rapid deployment of international tele-intensive care unit services in War-Torn Syria. Ann Am Thorac Soc. 2016;13(2):165–72. 26788827 10.1513/AnnalsATS.201509-589OT PMC5461955

[10] Agbali RA, Balas EA, Beltrame F, Heboyan V, De Leo G. A review of questionnaires used for the assessment of telemedicine. J Telemed Telecare. 2024;30(10):1636–66. 10.1177/1357633X231166161 37032470

[11] Hajesmaeel-Gohari S, Bahaadinbeigy K. The most used questionnaires for evaluating telemedicine services. BMC Med Inform Decis Mak. 2021;21(1):36. 33531013 10.1186/s12911-021-01407-y PMC7852181

[12] Yip MP, Chang AM, Chan J, MacKenzie AE. Development of the Telemedicine Satisfaction Questionnaire to evaluate patient satisfaction with telemedicine: a preliminary study. J Telemed Telecare. 2003;9(1):46–50. 12641893 10.1258/135763303321159693

[13] Bakken S, Grullon-Figueroa L, Izquierdo R, Lee NJ, Morin P, Palmas W, et al. Development, validation, and use of English and Spanish versions of the telemedicine satisfaction and usefulness questionnaire. J Am Med Inform Assoc. 2006;13(6):660–7. 16929036 10.1197/jamia.M2146 PMC1656962

[14] Buysse HE, Coorevits P, Van Maele G, Hutse A, Kaufman J, Ruige J, et al. Introducing telemonitoring for diabetic patients: development of a telemonitoring “Health Effect and Readiness” Questionnaire. Int J Med Inform. 2010;79(8):576–84. 20599161 10.1016/j.ijmedinf.2010.05.005

[15] Morgan DG, Kosteniuk J, Stewart N, O’Connell ME, Karunanayake C, Beever R. The telehealth satisfaction scale: reliability, validity, and satisfaction with telehealth in a rural memory clinic population. Telemed J E Health. 2014;20(11):997–1003. 25272141 10.1089/tmj.2014.0002 PMC4457516

[16] Parmanto B, Lewis AN Jr, Graham KM, Bertolet MH. Development of the Telehealth Usability Questionnaire (TUQ). Int J Telerehabil. 2016;8(1):3–10. 27563386 10.5195/ijt.2016.6196 PMC4985278

[17] Peacock JL, Peacock PJ. Oxford Handbook of Medical Statistics 2e. Oxford: Oxford University Press; 2020. 10.1093/med/9780198743583.001.0001.

[18] Bonett DG. Sample size requirements for testing and estimating coefficient alpha. J Educ Behav Stat. 2002;27(4):335–40.

[19] Feldt LS, Woodruff DJ, Salih FA. Statistical inference for coefficient alpha. Appl Psychol Meas. 1987;11(1):93–103.

[20] Bühner M. Einführung in die Test- und Fragebogenkonstruktion. 3., aktualisierte und erweiterte Auflage, ed. Pearson Deutschland GmbH; 2011.

[21] Bland JM, Altman DG. Statistics notes: validating scales and indexes. BMJ. 2002;324(7337):606–7. 11884331 10.1136/bmj.324.7337.606 PMC1122519

[22] Brooke J. SUS: A quick and dirty usability scale. Usability Eval Ind. Boca Raton: CRC Press. 1995. p. 189–94.

[23] Shang Z. Use of Delphi in health sciences research: a narrative review. Medicine (Baltimore). 2023;102(7):e32829. 36800594 10.1097/MD.0000000000032829 PMC9936053

[24] Diamond IR, Grant RC, Feldman BM, Pencharz PB, Ling SC, Moore AM, et al. Defining consensus: a systematic review recommends methodologic criteria for reporting of Delphi studies. J Clin Epidemiol. 2014;67(4):401–9. 24581294 10.1016/j.jclinepi.2013.12.002

[25] Lewin SR, Attoye T, Bansbach C, Doehle B, Dube K, Dybul M, et al. Multi-stakeholder consensus on a target product profile for an HIV cure. Lancet HIV. 2021;8(1):e42–50. 33271125 10.1016/S2352-3018(20)30234-4 PMC7773628

[26] Meyer VM, Benjamens S, Moumni ME, Lange JFM, Pol RA. Global overview of response rates in patient and health care professional surveys in surgery: a systematic review. Ann Surg. 2022;275(1):e75–81. 32649458 10.1097/SLA.0000000000004078 PMC8683255

[27] Wu MJ, Zhao KLY, Fils-Aime F. Response rates of online surveys in published research: a meta-analysis. Comput Hum Behav Rep. 2022;7:100206.

[28] Story DA, Tait AR. Survey research. Anesthesiology. 2019;130(2):192–202. 30688782 10.1097/ALN.0000000000002436

[29] Ritter PL, Stewart AL, Kaymaz H, Sobel DS, Block DA, Lorig KR. Self-reports of health care utilization compared to provider records. J Clin Epidemiol. 2001;54(2):136–41. 11166528 10.1016/s0895-4356(00)00261-4

[30] Boyle WA, Palmer CM, Konzen L, Fritz BA, White J, Simkins M, et al. Telemedicine critical care-mediated mortality reductions in lower-performing patient diagnosis groups: a prospective, before and after study. Crit Care Explor. 2023;5(10):e0979. 37753237 10.1097/CCE.0000000000000979 PMC10519574

[31] Breslow MJ, Rosenfeld BA, Doerfler M, Burke G, Yates G, Stone DJ, et al. Effect of a multiple-site intensive care unit telemedicine program on clinical and economic outcomes: an alternative paradigm for intensivist staffing. Crit Care Med. 2004;32(1):31–8. 14707557 10.1097/01.CCM.0000104204.61296.41

[32] Lilly CM, Cody S, Zhao H, Landry K, Baker SP, McIlwaine J, et al. Hospital mortality, length of stay, and preventable complications among critically ill patients before and after tele-ICU reengineering of critical care processes. JAMA. 2011;305(21):2175–83. 21576622 10.1001/jama.2011.697

[33] Lilly CM, McLaughlin JM, Zhao H, Baker SP, Cody S, Irwin RS, et al. A multicenter study of ICU telemedicine reengineering of adult critical care. Chest. 2014;145(3):500–7. 24306581 10.1378/chest.13-1973

[34] Sadaka F, Palagiri A, Trottier S, Deibert W, Gudmestad D, Sommer SE, et al. Telemedicine intervention improves ICU outcomes. Crit Care Res Pract. 2013;2013:456389. 23365729 10.1155/2013/456389 PMC3556431

[35] Willmitch B, Golembeski S, Kim SS, Nelson LD, Gidel L. Clinical outcomes after telemedicine intensive care unit implementation. Crit Care Med. 2012;40(2):450–4. 22020235 10.1097/CCM.0b013e318232d694

[36] Coletti C, Elliott DJ, Zubrow MT. Resident perceptions of a tele-intensive care unit implementation. Telemed J E Health. 2010;16(8):894–7. 20925564 10.1089/tmj.2010.0040

[37] Ward MM, Ullrich F, Potter AJ, MacKinney AC, Kappel S, Mueller KJ. Factors affecting staff perceptions of Tele-ICU service in Rural Hospitals. Telemed J E Health. 2015;21(6):459–66. 25734922 10.1089/tmj.2014.0137

[38] WHO WHO. Framework for the Implementation of a Telemedicine Service. 2016. p. 45–6.

[39] Venkataraman A, Fatma N, Edirippulige S, Ramamohan V. Facilitators and barriers for telemedicine systems in India from multiple stakeholder perspectives and settings: a systematic review. Telemed J E Health. 2024;30(5):1341–56. 38206654 10.1089/tmj.2023.0297

[40] Agha Z, Schapira RM, Laud PW, McNutt G, Roter DL. Patient satisfaction with physician-patient communication during telemedicine. Telemed J E Health. 2009;15(9):830–9. 19919189 10.1089/tmj.2009.0030

