# Supplementary material for: TICU-Feedback-Tool: development and pilot application of a questionnaire to assess performance in tele-intensive care collaborations
Source: BMC Health Serv Res. 2025 Mar 20;25:412. doi: 10.1186/s12913-025-12565-4 (PMC11924688; doi:10.1186/s12913-025-12565-4)
Supplement: Supplementary file 1 — Supplementary Material 1. [file 12913_2025_12565_MOESM1_ESM.docx]

Supplement 1 (Table S1): Pre-selection and consensus of TICU-Feedback-Tool indicators (n=20)

|  |  |  | Importance High % | Reliability for true self-report High % |
| --- | --- | --- | --- | --- |
|  |  |  |  |  |
| User-friendliness | System usability Scale (SUS) | 1. I think I would like to use this system/tele-medical device frequently | 95 | 95 |
|  |  | 1. I found the system/tele-medical device unnecessarily complex | 55 | 70 |
|  |  | 1. I thought the system/tele-medical device was easy to use | 90 | 80 |
|  |  | 1. I think that I would need the support of a technical person to be able to use this system | 70 | 80 |
|  |  | 1. I found the various functions in this system/ tele-medical device were well integrated | 65 | 75 |
|  |  | 1. I thought there was too much inconsistency in this system | 45 | 60 |
|  |  | 1. I would imagine that most people would learn to use this system/the tele-medical device very quickly | 70 | 70 |
|  |  | 1. I found the system very cumbersome to use | 35 | 50 |
|  |  | 1. I felt very confident using the system/tele-medical device | 85 | 85 |
|  |  | 1. I needed to learn a lot of things before I could get going with this system/ tele-medical device | 55 | 55 |
| Subjective benefit assessment and usabillity | Subjective benefit assessment: benefit for patients | 1. Patient safety was improved/increased by the tele-medical visits | 100 | 75 |
|  |  | 1. The patients' quality of care was improved by the tele-medical visits | 95 | 80 |
|  |  | 1. The telemedical visits positively contributed to error avoidance/error prevention in the treatment of my patients | 80 | 55 |
|  | Subjective benefit assessment: benefit for medical staff (physicians and nurses) | 1. I felt well supported in taking difficult decisions by tele-medical visits | 95 | 95 |
|  |  | 1. There was always enough time to discuss my concerns during tele-medical visits | 85 | 85 |
|  |  | 1. If necessary, telemedical visits were arranged at short notice | 90 | 95 |
|  |  | 1. By taking part in tele-medical visit, I was able to refresh or acquire important medical knowledge | 75 | 60 |
|  | Trust & Working Relationship | 1. I was always able to address any uncertainties or treatment errors openly | 80 | 50 |
|  |  | 1. I implemented the treatment plans as discussed in tele-medical visits | 90 | 60 |
|  |  | 1. The collaboration with the tele-medical specialist was always friendly and constructive | 95 | 65 |
|  | Interaction with patients | 1. During the rounds, the tele-medical specialist treated my patients respectfully | 90 | 80 |
|  |  | 1. My patients accepted the tele-medical specialist very well | 80 | 65 |
|  | Technical performance | 1. The picture quality was good and error free | 95 | 90 |
|  |  | 1. The sound quality was good and error-free | 95 | 95 |
|  |  | 1. From the technical side communication with the tele-medical physician functioned well | 85 | 85 |
|  | Overall | 1. The tele-medical rounds helped me in treating my patients | 90 | 95 |
| Acceptance and Improvement | Attitude of the "users" towards tele-medical rounds | 1. Initially, I had reservations about conducting tele-medical visits with the telemedical device | 50 | 55 |
|  |  | 1. In principle, I have a positive attitude towards tele-medical consultations using the device | 75 | 65 |
|  |  | 1. I am fundamentally opposed to tele-medical consultations via the device | 45 | 55 |
|  |  | 1. I have ethical concerns about tele-medical visits | 60 | 70 |
|  |  | 1. I have data protection concerns about tele-medical visits | 70 | 80 |
|  |  | 1. Overall, I am satisfied with tele-medical rounds | 75 | 75 |
|  |  | 1. All in all, I think the tele-medical visits work well | 85 | 70 |
|  |  | 1. All in all, I think there is still potential for quality improvement of the tele-medical visits | 75 | 60 |
|  | Potential for improvement | 1. What points would you like to change about the tele-medical visits for further improvement? | 85 | 75 |

*Indicators excluded by consensus survey with approval <75% for high importance
*Indicators exceptionally included in pilot version of the tool despite approval <75% for high importance
