# Supplementary material for: TICU-Feedback-Tool: development and pilot application of a questionnaire to assess performance in tele-intensive care collaborations
Source: BMC Health Serv Res. 2025 Mar 20;25:412. doi: 10.1186/s12913-025-12565-4 (PMC11924688; doi:10.1186/s12913-025-12565-4)
Supplement: Supplementary file 2 — Supplementary Material 2. [file 12913_2025_12565_MOESM2_ESM.docx]

Supplement 2: Written instructions for all participants before entering the consensus survey

Dear colleagues,

You are experienced intensivists who have gained extensive expertise in tele-intensive care collaboration in recent years. Tele-ICU networks offer the opportunity for (inter)national cooperation between different hospitals with heterogeneous conditions in patient care. We aim to develop two self-reporting online questionnaires for quality management and needs-adaptive tele-medical interventions.

Firstly, we would like to evaluate the quality of our tele-consultations using a feedback questionnaire. Secondly, we aim to develop a questionnaire that captures the structures and processes of hospitals in general, intensive care units in particular, and infection prevention and control management, in order to tailor our consultations according to specific needs.

We kindly request your support and collaboration in developing the two online questionnaires. In the link below, you will find a survey regarding the planned questions for both surveys. We kindly ask you to assess the importance of each question regarding intensive care work („Importance high vs. low“). We also ask you to assess the reliability of each question for true self-report (“Reliability for true self-report high vs low“) i.e. do you think this question will be answered truthfully.

Please note that the questionnaires should be suitable for international cooperation, meaning that they include questions that may not be equally relevant in all countries, such as malaria diagnostics or mass casualty management.

In a nutshell:

Feedback Questionnaire: Which questions do we need to ask to evaluate the satisfaction with tele-medical consultations and the (technical) user-friendliness of the system?

Structural Questionnaire: Which questions do we need to ask about the structures of the collaborating hospitals / intensive care units, to be able to provide effective clinical consultations and potentially conduct additional training according to the needs?

We highly appreciate your participation and value the input of as many colleagues as possible in developing these questionnaires.

Thank you very much!
