# Supplementary material for: TICU-Feedback-Tool: development and pilot application of a questionnaire to assess performance in tele-intensive care collaborations
Source: BMC Health Serv Res. 2025 Mar 20;25:412. doi: 10.1186/s12913-025-12565-4 (PMC11924688; doi:10.1186/s12913-025-12565-4)
Supplement: Supplementary file 3 — Supplementary Material 3. [file 12913_2025_12565_MOESM3_ESM.docx]

Supplement 3 (Table S2): Pilot version of TICU-Feedback-Tool

|  |  |  | Strongly disagree | Disagree | Neutral | Agree | Strongly agree |
| --- | --- | --- | --- | --- | --- | --- | --- |
|  |  |  | 1 | 2 | 3 | 4 | 5 |
| User-friendliness | System usability Scale (SUS) | 1. I think I would like to use this system/tele-medical device frequently |  |  |  |  |  |
|  |  | 1. I thought the system/tele-medical device was easy to use |  |  |  |  |  |
|  |  | 1. I felt very confident using the system/tele-medical device |  |  |  |  |  |
| Subjective benefit assessment and usabillity | Subjective benefit assessment: benefit for patients | 1. Patient safety was improved/increased by the tele-medical visits |  |  |  |  |  |
|  |  | 1. The patients' quality of care was improved by the tele-medical visits |  |  |  |  |  |
|  |  | 1. The telemedical visits positively contributed to error avoidance/error prevention in the treatment of my patients |  |  |  |  |  |
|  | Subjective benefit assessment: benefit for medical staff (physicians and nurses) | 1. I felt well supported in taking difficult decisions by tele-medical visits |  |  |  |  |  |
|  |  | 1. There was always enough time to discuss my concerns during tele-medical visits |  |  |  |  |  |
|  |  | 1. If necessary, telemedical visits were arranged at short notice |  |  |  |  |  |
|  |  | 1. By taking part in tele-medical visit, I was able to refresh or acquire important medical knowledge |  |  |  |  |  |
|  | Trust & Working Relationship | 1. I was always able to address any uncertainties or treatment errors openly |  |  |  |  |  |
|  |  | 1. I implemented the treatment plans as discussed in tele-medical visits |  |  |  |  |  |
|  |  | 1. The collaboration with the tele-medical specialist was always friendly and constructive |  |  |  |  |  |
|  | Interaction with patients | 1. During the rounds, the tele-medical specialist treated my patients respectfully |  |  |  |  |  |
|  |  | 1. My patients accepted the tele-medical specialist very well |  |  |  |  |  |
|  | Technical performance | 1. The picture quality was good and error free |  |  |  |  |  |
|  |  | 1. The sound quality was good and error-free |  |  |  |  |  |
|  |  | 1. From the technical side communication with the tele-medical physician functioned well |  |  |  |  |  |
|  | Overall | 1. The tele-medical rounds helped me in treating my patients |  |  |  |  |  |
| Acceptance and Improvement | Attitude of the "users" towards tele-medical rounds | 1. In principle, I have a positive attitude towards tele-medical consultations using the device |  |  |  |  |  |
|  |  | 1. I have ethical concerns about tele-medical visits |  |  |  |  |  |
|  |  | 1. I have data protection concerns about tele-medical visits |  |  |  |  |  |
|  |  | 1. Overall, I am satisfied with tele-medical rounds |  |  |  |  |  |
|  |  | 1. All in all, I think the tele-medical visits work well |  |  |  |  |  |
|  |  | 1. All in all, I think there is still potential for quality improvement of the tele-medical visits |  |  |  |  |  |
|  | Potential for improvement | 1. What points would you like to change about the tele-medical visits for further improvement? |  | | | | |
